# Metal pollution in the topsoil of lands adjacent to Sahiwal Coal Fired Power Plant (SCFPP) in Sahiwal, Pakistan

**DOI:** 10.1371/journal.pone.0298433

**Published:** 2024-02-15

**Authors:** Muhammad Luqman, Aliza Niazi, Saif Ur Rehman Kashif, Fariha Arooj, Syed Aziz ur Rehman, Muhammad Umer Farooq Awan, Muhammad Wasim, Faakhar Raza

**Affiliations:** 1 Department of Environmental Sciences, University of Veterinary & Animal Sciences (UVAS) Lahore, Pakistan; 2 Department of Botany, Government College University, Lahore, Pakistan; 3 Institute of Biochemistry and Biotechnology, University of Veterinary & Animal Sciences (UVAS) Lahore, Pakistan; 4 Pakistan Council of Research in Water Resources (PCRWR), Regional Office, Lahore, Pakistan; BOKU: Universitat fur Bodenkultur Wien, AUSTRIA

## Abstract

Coal fly ash from a coal fired power plant is a significant anthropogenic source of various heavy metals in surrounding soils. In this study, heavy metal contamination in topsoil around Sahiwal coal fired power plant (SCFPP) was investigated. Within distance of 0–10, 11–20, 21–30 and 31–40 km of SCFPP, total 56 soil samples were taken, 14 replicate from each distance along with a background subsurface soil sample beyond 60 km. Soil samples were subjected to heavy metals analysis including Fe, Cu and Pb by Atomic Absorption Spectrophotometer (AAS). Composite samples for each distance were analyzed for Al, As, Ba, Cd, Co, Cr, Mn, Mo, Ni, Se, Sr, Zn by Inductively Coupled Plasma (ICP). Pollution indices of exposed soil including Enrichment Factor (EF), Contamination Factor (CF), Geoaccumulation Index (I_geo_), and Pollution Load Index (PLI) were calculated. Ecological risk index ($E_{r}^{i}$) of individual metals and the Potential Ecological Risk Index (PERI) for all metals were determined. Soil samples within 40 km of SCFPP were significantly polluted with Pb (mean 2.81 ppm), Cu (mean 0.93 ppm), and Fe (mean 7.93 ppm) compared to their background values (Pb 0.45, Cu 0.3, and Fe 4.9 ppm). Some individual replicates were highly contaminated where Pb, Fe, and Cu values were as high as 6.10, 35.4 and 2.51 ppm respectively. PLI, I_geo_, CF, and EF for metals classified the soil around CFPP as “moderate to high degree of pollution”, “uncontaminated to moderately contaminated”, “moderate to very high contamination”, and “moderate to significant enrichment” respectively with average values for Cu as 2.75, 0.82, 3.09, 4.01; Pb 4.79, 1.56, 6.16, 7.76, and for Fe as 1.20, 0.40, 1.62, 3.35 respectively. Average Ecological Risk Index ($E_{r}^{i}$) of each metal and Potential Ecological Risk Index (PERI) for all metals classified the soils as “low risk soils” in all distances. However, ($E_{r}^{i}$) of Pb at a number of sites in all distances have shown “moderate risk”. The linear correlation of physico-chemical parameter (EC, pH, Saturation %) and metals have recorded several differential correlations, however, their collective impact on Pb in 0–10 km, has recorded statistically significant correlation (p-value 0.01). This mix of correlations indicates complex interplay of many factors influencing metal concentrations at different sampling sites. The concentration of As, Cr, Co, Cd, and Zn was found within satisfactory limits and lower than in many parts of the world. Although the topsoil around SCFPP is largely recorded at low risk, for complete assessment of its ecological health, further research considering comprehensive environmental parameters, all important trace metals and variety of input pathways is suggested.

## Introduction

Thermal power from burning fossil fuel especially coal is the most renowned source of energy production in the underdeveloped countries. However, development of energy projects contaminates the environment including water, air, and soil, which act as a sink. Heavy metals contamination is a major concern linked with coal fired power plant (CFPP). Despite the incremental increase in use of renewable energy resources around the world, till 2022, the use of coal for power generation was increasing. However, its use is expected to decrease in future. Global coal supply in 2015 was 29% of the energy that increased to 37% in 2020, but it is forecasted that it will be about 24% by the 2035 [1]. Coal is a major and relatively cheaper energy source found in the earth crust, hence a preferred choice for developing nations with little resources. Coal is a sedimentary rock that is brownish black in color and clayey in its texture [2]. Some researchers have classified elements of coal into three groups as nonvolatile elements (Ca, Al, Si, Fe, Hf, Th, etc.), volatile elements (Cd, As, Pb, Cu, Zn, Mo, etc.), and very volatile elements (Halogens, N, B, Hg, S, Se) [3]. After the burning of coal in coal fired power plant, various combustion residues are produced. Some of the combustion residues are boiler slag, bottom ash and fly ash [1]. The melted form of ash after coal burning is known as the boiler slag. It is usually settled down at the bottom of the boiler or bound to the exhaust in filters. Coal waste ash consist of almost 70% coal fly ash whereas the remaining are the bottom ash [1]. Coal bottom ash is a grain size fraction of coal combustion residue that is disposed of at the ash ponds in the form of slurry.

Coal combustion byproducts are of great concern especially when it comes to the coal fly ash. Coal fly ash, having a silt like appearance, is composed of sizes of about 0.5 to 200μm which remains suspended and fall out from the power plant [4]. The characteristics of coal fly ash depends upon the type of coal and its process of ignition [5]. Its combustion can lead to the release of carbon monoxide, sulfur dioxide, nitrogen oxide, and various heavy metals like Cu, Cd, Pb, Ca, Fe, Al, Na, K, Si and Ti into the environment [5, 6]. The heavy metals release from the CFPPs deposit them into the nearby terrestrial and aquatic environment, significantly affecting the quality of surrounding areas [7]. If it is disposed of in land it can be easily leached out and contaminate the nearby groundwater [4]. Such heavy metals can have drastic impacts on the ecological health.

Heavy metals can have serious negative impacts on environment, flora and fauna including humans [8]. Various studies have reported the association of coal fly ash with plant and animal health including genotoxicity, effected reproduction processes, and hematological and immune system changes [9]. It was observed that >90% of the cabbages had lead (Pb) and >30% had Arsenic (As) concentration exceeding the permissible limit near the CFPP [7]. Physiological systems (hematopoietic, central nervous, cardiovascular, urinary, gastrointestinal, and reproductive system) of exposed animals can get affected by coal ash toxicants like arsenic, cadmium, lead, and mercury [10]. High levels of heavy metals like chromium, selenium, zinc, and copper can also have various impacts on the hematopoietic, gastrointestinal, and reproductive systems on animals including human [10]. Soil is the final sink for environmental contaminations like heavy metals get scattered and dispersed in the natural environment by various anthropogenic activities. Although, coal fly ash enhances the edaphic characteristics of soil, it can affect the soil and the environment by introducing toxic metals like Cu, Pb, Cr, and As [5]. Impacts of environmental contaminant in soil vary from site to site and depend upon soil texture, humidity, density, absorption properties and several other factors [11]. Crops grown on such polluted soils may lead to serious health conditions like kidney malfunctioning, lung cancer, and fractures in bones in exposed humans. Impairments of liver, endocrine and nervous system are also reported health conditions linked with heavy metals [12]. Several studies have reported the association between coal fly ash exposure and human health conditions in many parts of the world except Pakistan. They have reported the link of fly ash with chronic cough, lung cancer, obstructive pulmonary disease, insomnia, and various cardiovascular problems [9]. Apart from health impacts on humans, such heavy metal contamination impacts flora and fauna.

Millions of hectares of land contaminated with trace metals need remediation to get rid of metals like Cu, Ni, Zn, As, Se, Cd, Co, Hg, and Pb [13, 14]. Various remediation techniques are applied ex-situ and in-situ to remove metal contamination from soils [15–17]. These include landfilling [18, 19], soil washing [20], soil sealing or encapsulation [21], surface capping [22] solidification [23], electro-kinetic extraction [24], verification [18], stabilization [23], bioremediation [18], and phytoremediation [25]. All these soil remediation techniques are successful to varying degrees and selection of technique depend upon several factors including location, geography of site, soil structure, nature of the metal involved, extent of contamination, regulations of the land, available budget and available time [26]. Each of this technique involves physical, biological, chemical or electrical process to either contain or immobilize or extract the heavy metals from the contaminated soil. Among these, most promising and cost effective technique is phytoremediation that is also environment friendly and has more public acceptability. Although this technique is time consuming and less efficient currently, but is a good techniques to extract metals from contaminated soils at shallow depths. About 400 hyper-accumulator plant species are tested and applied for bioremediation of contaminated soils in the laboratory and field [27]. The limitation of majority of these species is slow growth, lower biomass and limited ability for hyper-accumulation [27]. Laboratory scale and field level efforts are being made by researchers to enhance these traits. Some species like Hemp (Cannabis sativa L.) are reported to have high biomass, fast growing, and good hyper-accumulator for metals like Cr, Pb, Zn, Ni, and Cd, hence can be ideal candidate for bioremediation [27–29].

Several studies have linked the coal fly ash with impaired conditions of flora and fauna around coal fired power plants. Health of fish and wildlife has been reported to be compromised due to Selenium accumulation released from coal fly ash from Rampal power plant, India [30]. Another study reported the hematological changes in raccoons due to heavy metal exposure from coal fly ash in United States [10]. Coal fly ash generated from coal power plant has started to gain a potentially significant anthropogenic source of Arsenic (As). In India, coal fly ash residue is causing serious environmental problems by elevating the trace amount of the metals like Arsenic [31]. Similarly, study conducted to assess the Chinese Coal fired power plant’s soil heavy metal contamination shows. Ecological damages and high risks are reported to be linked with coal fly ash induced presence of mercury, arsenic, cadmium, and some other elements in the soil due to Chinese Coal fired power plant [32]. The soil around Russian Novocherkassk power plant was found rich in cadmium, nickel, and lead and an increased risk of carcinogenicity for humans is warned [6]. Heavy metal pollution due to coal fired power plants is feared to increase in various underdeveloped countries like Pakistan, which are setting up CFPP to overcome energy crisis.

Pakistan has about 185.175 tons of coal resources [33], yet has few coal fired power plants and largely rely on imported coal. In the past couple of decades, the country has faced severe energy crisis, which compelled the authorities to build thermal power plants including coal fired power plants in the country on urgent basis. Pakistan has built two large CFPP of 1320 MW capacity each in Port Qasim, Sindh and Sahiwal, Punjab. SCFPP is one of the two huge coal fired power plants of Pakistan, which uses imported bituminous coal from South Africa and Indonesia. Stack height of SCFPP is about 182 meter and uses the super critical burning technology for coal. It is operational since October, 2017, and consumes about 4.48 million tons of coal every year for 22 hours daily [2]. Pakistan lacks research regarding the heavy metal content present in coal fly ash and its impacts on soil, water, flora, fauna and humans. Current study was conducted to quantify heavy metal contents in nearby soils and to assess health impacts of coal fly ash on residents around SCFFP.

## Materials and methods

### Study area

**Fig 1:**
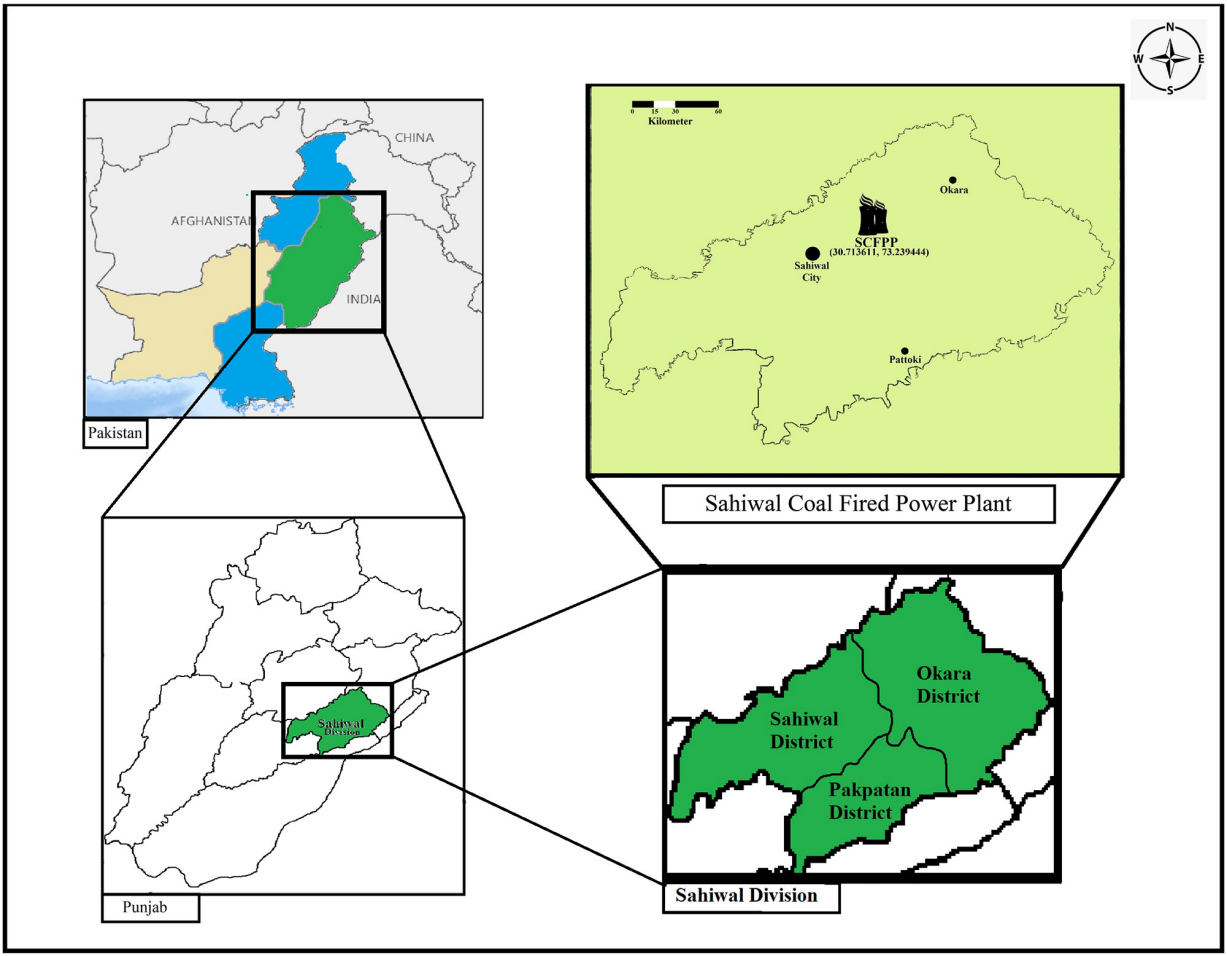
Location of Sahiwal Coal Fired Power Plant (SCFPP) (30.713611, 73.2394444).

On fertile agricultural lands of Qadirabad Village, about 15 km in the northeast of Sahiwal City, the Sahiwal coal fired power plant (SCFPP) is located. It is situated at the 30° 42′ 49″ N, 73° 14′ 22″ E of Pakistan (Fig 1). The total capacity of the power plant is about 1320 Mega Watt (MW). The divisional headquarter of Sahiwal city accommodates large population of about 2.51 million, which is under the influence of coal fly ash from this plant.

### Soil sample collection

**Fig 2:**
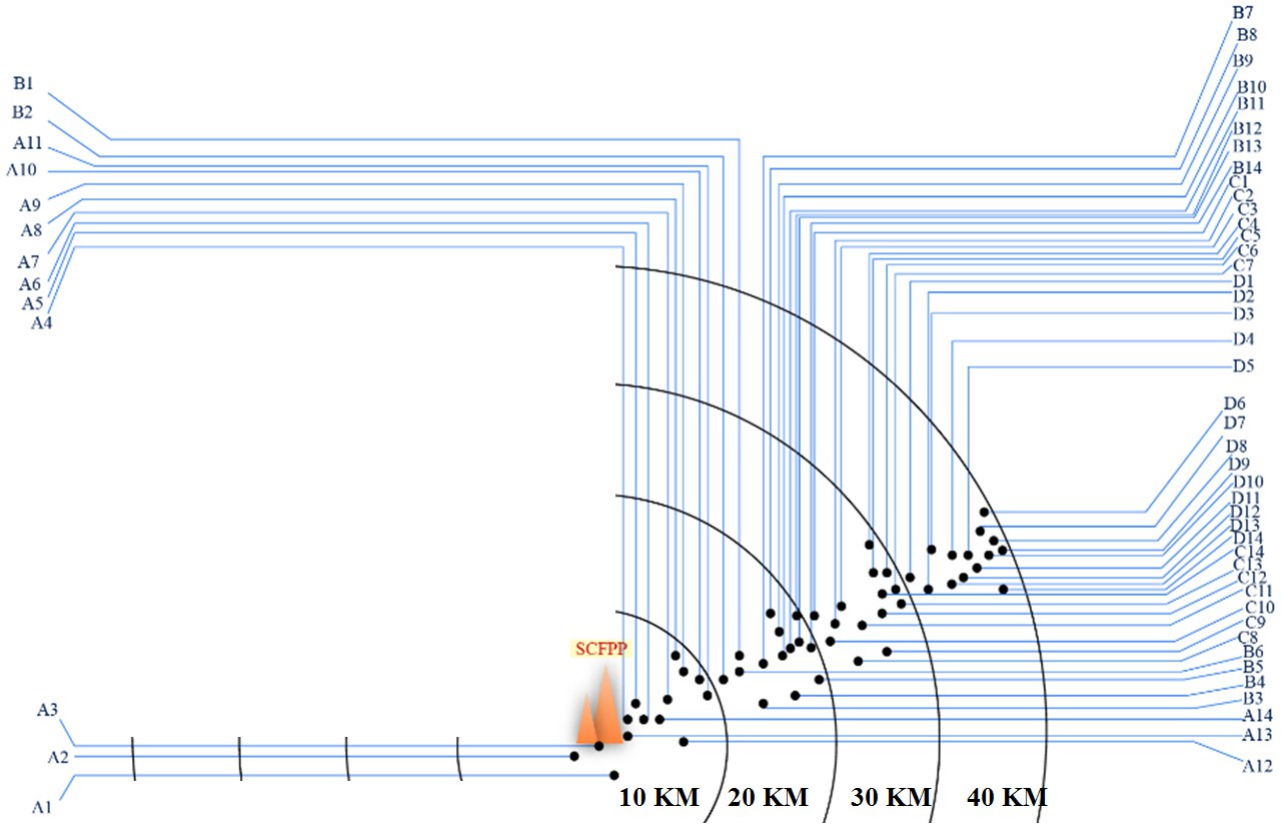
Sampling sites within 10 km, 20 km, 30 km, 40 km distance of SCFPP (sampling coordinates details in Table 1).

Total 56 topsoil samples were collected within 10 km, 20 km, 30 km, and 40 km distance of Sahiwal coal fired power plant. From each distance category, 14 replicates samples were collected. A background reference soil sample was also collected beyond 60 km from Sahiwal coal fired power plant (Fig 2), (Table 1). The soil samples were collected by throwing quadrates (2 ft^2^) on undisturbed land and collecting upper 10 cm soil by using a spade. Location of the sample sites were recorded by using Garmin Oregon 750 t GPS unit. Each soil sample was about 1.5 kg in weight which was put in a labeled, clean polythene bag. Samples were later transported to the UVAS laboratory for the analysis of heavy metals.

**Table 1:** Sampling coordinates.

Distance	Replicate No.	Decimal Degrees (DD)	Degrees, Minutes, and Seconds (DMS)
**10 km**	A1	30.7048395, 73.2401083	30° 42’ 17.424"N 73° 14’ 24.3888"E
	A2	30.7055613, 73.2425951	30° 42’ 20.019"N 73° 14’ 33.3414"E
	A3	30.7081669, 73.2478321	30° 42’ 29.4006"N 73° 14’ 52.1952"E
	A4	30.7184245, 73.2585214	30° 43’ 6.3294"N 73° 15’ 30.6756"E
	A5	30.7168606, 73.2584201	30° 43’ 0.6996"N 73° 15’ 30.312"E
	A6	30.7172431, 73.2587681	30° 43’ 2.0742"N 73° 15’ 31.5648"E
	A7	30.7166153, 73.2581335	30° 42’ 59.814"N 73° 15’ 29.2818"E
	A8	30.717982, 73.2612160	30° 43’ 4.7346"N 73° 15’ 40.3776"E
	A9	30.7187498, 73.2627204	30° 43’ 7.5", 73°N 15’ 45.792"E
	A10	30.7197028, 73.2611650	30° 43’ 10.9302"N 73° 15’ 40.194"E
	A11	30.721273, 73.267317	30° 43’ 16.5828"N 73° 16’ 2.3412"E
	A12	30.721443, 73.266968	30° 43’ 17.1948"N 73° 16’ 1.0848"E
	A13	30.7200098, 73.2618634	30° 43’ 12.0354"N 73° 15’ 42.7068"E
	A14	30.7201616, 73.2623760	30° 43’ 12.5826"N 73° 15’ 44.5536"E
**20 km**	B1	30.7887168, 73.4087811	30° 47’ 19.3806"N 73° 24’ 31.6116"E
	B2	30.7874324, 73.4086856	30° 47’ 14.7546"N 73° 24’ 31.2696"E
	B3	30.753889, 73.339722	30° 45’ 14.0004"N 73° 20’ 22.9986"E
	B4	30.754722, 73.34	30° 45’ 16.9992"N 73° 20’ 24"E
	B5	30.754444, 73.340278	30° 45’ 15.9978"N 73° 20’ 25.0002"E
	B6	30.754444, 73.340556	30° 45’ 15.9978"N 73° 20’ 26.0016"E
	B7	30.754722, 73.340278	30° 45’ 16.9992"N 73° 20’ 25.0002"E
	B8	30.755, 73.341389	30° 45’ 17.9994"N 73° 20’ 29.0004"E
	B9	30.755, 73.341111	30° 45’ 17.9994"N 73° 20’ 27.999"E
	B10	30.755278, 73.341389	30° 45’ 19.0008"N 73° 20’ 29.0004"E
	B11	330.754722, 73.340556	30° 45’ 16.9992"N 73° 20’ 26.0016"E
	B12	30.755833, 73.341389	30° 45’ 20.9982"N 73° 20’ 29.0004"E
	B13	30.756111, 73.341667	30° 45’ 21.9996"N 73° 20’ 30.0012"E
	B14	30.756111, 73.341111	30° 45’ 21.9996"N 73° 20’ 27.999"E
**30 km**	C1	30.825278, 73.486944	30° 49’ 31.0008"N 73° 29’ 12.9978"E
	C2	30.824167, 73.4875	30° 49’ 27.0006"N 73° 29’ 14.9994"E
	C3	30.791944, 73.431667	30° 47’ 30.9984"N 73° 25’ 54.0012"E
	C4	30.791667, 73.431944	30° 47’ 30.0012"N 73° 25’ 54.9984"E
	C5	30.791667, 73.432778	30° 47’ 30.0012"N 73° 25’ 58.0002"E
	C6	30.791667, 73.433611	30° 47’ 30.0012"N 73° 26’ 0.999"E
	C7	30.791389, 73.433611	30° 47’ 28.9998"N 73° 26’ 0.999"E
	C8	30.790556, 73.433889	30° 47’ 26.001"N 73° 26’ 1.9998"E
	C9	30.791111, 73.433333	30° 47’ 27.9996"N 73° 25’ 59.9988"E
	C10	30.791667, 73.433333	30° 47’ 30.0012"N 73° 25’ 59.9988"E
	C11	30.790556, 73.432778	30° 47’ 26.001"N 73° 25’ 58.0002"E
	C12	30.790833, 73.433889	30° 47’ 26.9982"N 73° 26’ 1.9998"E
	C13	30.790556, 73.433056	30° 47’ 26.001"N 73° 25’ 59.001"E
	C14	30.791389, 73.433333	30° 47’ 28.9998"N 73° 25’ 59.9988"E
**40 km**	D1	30.8671778, 73.5900535	30° 52’ 1.8402"N 73° 35’ 24.1938"E
	D2	30.8671760, 73.5903824	30° 52’ 1.8336"N 73° 35’ 25.3752"E
	D3	30.845556, 73.527778	30° 50’ 44.001"N 73° 31’ 40.0002"E
	D4	30.845556, 73.5275	30° 50’ 44.001"N 73° 31’ 39" E
	D5	30.845278, 73.528611	30° 50’ 43.0008"N 73° 31’ 42.999"E
	D6	30.845556, 73.528611	30° 50’ 44.001"N 73° 31’ 42.999"E
	D7	30.845833, 73.529167	30° 50’ 44.9982"N 73° 31’ 45.0012"
	D8	30.846111, 73.529722	30° 50’ 45.9996"N 73° 31’ 46.9992"E
	D9	30.846667, 73.529444	30° 50’ 48.0012"N 73° 31’ 45.9978"E
	D10	30.846944, 73.53	30° 50’ 48.9984"N 73° 31’ 48"E
	D11	30.847222, 73.530278	30° 50’ 49.9986"N 73° 31’ 49.0002"E
	D12	30.845278, 73.528333	30° 50’ 43.0008"N 73° 31’ 41.9988"E
	D13	30.845278, 73.528056	30° 50’ 43.0008"N 73° 31’ 41.0016"E
	D14	30.846389, 73.529167	30° 50’ 46.9998"N 73° 31’ 45.0012"E
**Beyond 60 km**	S1	31.207222, 73.965	31° 12’ 25.9992"N 73° 57’ 54"E

### Soil sample preparation and extraction

Soil samples were dried under the sun by spreading on plastic trays. Dried samples of soil were homogenized by using mortal and pastel and passed through 2 mm sieve. Then the samples were further dried in hot oven at 105°C for about 24 hours to remove the remaining moisture content [2]. The soil samples were again homogenized.

The soil sample extraction was done by following the Diethylenetriaminepentaacetic acid micronutrient extraction method (DTPA method) [34]. 0.005 M DTPA (Diethylenetriaminepentaacetic acid), 0.01 M CaCl_2_.2H_2_O (Calcium chloride dihydrate) and 0.1 M TEA (triethanolamine) were taken. Three separate solutions of 1.97 g of DTPA, 1.47 g of CaCl_2_.2H_2_O, and 14.92 ml of TEA were made in three separate beakers by adding some distilled water. After that these three solutions were combined and volume was made up to 1 liter by adding distilled water. The pH of the above DTPA solution was adjusted to 7.3 by using 1.1 M HCL (Hydrochloric acid).

A 25 g of soil sample was taken in a 100 ml beaker, added 50 ml of DTPA solution, shaken for two hours, and filtered into the sample bottles and run on atomic absorption spectrophotometer (Hitachi Z-8230). The process was repeated for all replicate samples and background reference soil sample. Prior to sample, a blank and five standards were run on atomic absorption spectrophotometer (AAS) to adjust the standard curve for each element under study. The processed soil samples were analyzed for heavy metals including Cu, Fe and Pb. Composite Samples of each distance category were also prepared and extracted by using same DTPA method. The extracts of these composite samples were run on Inductively coupled plasma (Agilent 5110 ICP-OES) for heavy metals including Al, As, Ba, Cd, Co, Cr, Mn, Mo, Ni, Se, Sr, Zn.

Electrical conductivity (EC), and pH of soil was measured by Hanna Bench-top EC and pH meters. Saturation percentage and texture class of the soil samples was also determined. Textural class of the soil was determined based on the saturation percentage following bellow cutline values.

Saturation % <20 = Sandy soil
Saturation % 20–30 = Sandy loam soil
Saturation % 31–45 = Loam soil
Saturation % >45 = Clay soil

### Quantification of pollution

To quantify soil heavy metals contamination level, Enrichment Factor, Contamination Factor, Geoaccumulation Index, and Pollution Load Index parameters were used.

Enrichment Factor (EF) was calculated by using Eq I [35].

$$EF=(\frac{M}{Fe})sample/(\frac{M}{Fe})background$$ (I)

Here M is the concentration of element in soil sample. Iron was taken as a reference element as it has uniformed natural concentration and its geochemistry is similar to many other heavy metals. Background concentration of metal concerned was determined in the reference soil sample.

Soil was classified according to following Enrichment Factor (EF) categories.

Class 1: EF < 2 –Minimal Enrichment
Class 2: EF = 2–5 –Moderate Enrichment
Class 3: EF = 5–20 –Significant Enrichment
Class 4: EF = 20–40 –Very High Enrichment
Class 5: EF > 40 –Extremely High Enrichment

To assess the elemental pollution of soil, Geoaccumulation Index (I_geo_) was calculated by the Eq II [35].


$$I_{geo}={log}_{2}(\frac{{[M}_{sample}]}{{k[M}_{background}]})$$ (II)

Due to the presence of lithospheric effects in the background soil, k = 1.5 was taken as the correction factor. Background concentration of metal concerned was determined in the reference soil sample.

Soil was classified according to following Geoaccumulation Index categories.

Partially uncontaminated: 0 > I_geo_
Uncontaminated to moderately contaminated: 1 > I_geo_ > 0
Moderately contaminated: 2 > I_geo_ > 1
Moderately to heavily contaminated: 3 > I_geo_ > 2
Heavily Contaminated: 4 > I_geo_ > 3
Heavily to extremely contaminated: 5 > I_geo_ > 4
Extremely contaminated: I_geo_ > 5

Contamination Factor (CF) was calculated by using Eq III [35].


$$CF=\frac{{[M}_{sample}]}{{[M}_{background}]}$$ (III)

Background concentration of metal concerned was determined in the reference soil sample.

The contamination levels were classified into 1–6 scale based on their intensities.

Pollution load index (PLI) was obtained by taking the n^th^ root of total contamination factor product (nCF) [35] using Eq IV.


$$PLI={\left({CF}_{1}\times {CF}_{2}\times {CF}_{3}\ldots .\times {CF}_{n}\right)}^{1/n}$$ (IV)

Potential Ecological Risk Index (PERI) for overall risk of heavy metals under study was also determined using formula V [36].


$$PERI=\sum ({E_{r}^{i}}_{Fe}+{E_{r}^{i}}_{Cu}+{E_{r}^{i}}_{Pb})$$ (V)

$E_{r}^{i}$ is Ecological Risk Index for each metal and was calculated using following equation

$$E_{r}^{i}=Tr+CF$$ (VI)

Where,

CF = Contamination factor

Tr = Toxic Response Factor

Tr value used for Cu, Pb and Fe was 5, 5, and 1 respectively [37–39] Risk classification of $E_{r}^{i}$ and PERI is done according to following criteria [40, 41]

$E_{r}^{i}$ <40, PERI <150: Low Risk
$E_{r}^{i}$ 40 –<80, PERI 150 –<300: Moderate Risk
$E_{r}^{i}$ 80 –<160, PERI 300 –<600: High Risk
$E_{r}^{i}$ 160 –<320, PERI ≥600: Severe Risk
$E_{r}^{i}$ ≥320: Serious High Risk

### Statistical analysis

Mean, median, range, standard deviation and Analysis of Variance (ANOVA) were calculated for the concentration of heavy metals present in the samples of soil around SCFPP. Multiple and linear regression was performed between metal contents and physicochemical properties of soil.

## Results

Coal fly ash is contaminating soil with heavy metal pollution and can potentially impact human health negatively. All the heavy metals under study have shown greater concentrations in soils around SCFPP compared to distant background sample.

### Iron

Average iron concentration within 10 km of SCFPP was relatively higher (7.77 ppm) compared to distant soils in 11–20 Km (7.05) and 31–40 Km distance (6.01) (Table 2). However, in soils from 21–30 km distance the iron concentration was higher unexpectedly (10.88 ppm). Average Iron concentration within 40 km of SCFPP was 7.93 ppm, which is well above background subsurface soil sample value (4.9 ppm).

**Table 2:** Heavy metals concentration (ppm) in soils around SCFPP.

Replicates	within 10 km			Replicates.	11–20 km			Replicates	21–30 km			Replicates	31–40 km			Average 0–40 km		
	Fe	Cu	Pb		Fe	Cu	Pb		Fe	Cu	Pb		Fe	Cu	Pb	Fe	Cu	Pb
**A1**	18.9	0.49	3.3	B1	3.41	0.67	0.99	C1	5.27	1.62	1.26	D1	5.95	1.33	1.09	**7.93**	**0.93**	**2.81**
**A2**	19.2	2.42	4.2	B2	3.15	0.66	0.64	C2	1.79	0.5	0.54	D2	5.06	0.54	1.28			
**A3**	6.78	0.89	5.2	B3	3.91	0.68	1.02	C3	7.18	2.43	4	D3	3.98	0.67	6.1			
**A4**	2.47	0.27	0.78	B4	14.8	1.07	5.2	C4	15.3	1.12	6.1	D4	2.92	0.73	4			
**A5**	2.89	0.42	0.78	B5	5.89	0.84	1.34	C5	4.36	0.97	5	D5	3.72	0.82	5.4			
**A6**	2.08	0.2	0.47	B6	11.8	1.01	5	C6	15.5	1.42	4.5	D6	35.4	1.59	6.1			
**A7**	2.3	0.1	0.52	B7	4.63	0.81	0.9	C7	6.87	1.06	4.5	D7	6.07	0.84	1.23			
**A8**	32.8	2.26	3.5	B8	7.65	1.26	4	C8	5.64	0.58	1.28	D8	4.85	0.74	6.1			
**A9**	3.86	0.72	0.92	B9	3.46	0.64	0.97	C9	16.4	1.07	5.9	D9	6.24	0.96	4			
**A10**	3.65	0.69	1.26	B10	2.67	0.63	0.69	C10	7.74	1.55	5.7	D10	5.5	0.43	1.33			
**A11**	2.82	0.69	0.92	B11	8.4	1.02	4.7	C11	19.9	1.04	5	D11	1.12	0.5	0.83			
**A12**	2.72	0.8	0.98	B12	17.6	0.73	2.8	C12	10.8	2.51	5.9	D12	0.99	0.34	0.95			
**A13**	3.86	0.66	0.9	B13	6.78	0.85	0.45	C13	15.2	1.4	5.9	D13	1.11	0.39	1.37			
**A14**	4.38	0.71	0.99	B14	4.52	0.83	1.3	C14	20.4	1.22	6.1	D14	1.22	0.54	0.9			
**Mean**	7.77	**0.81**	1.77	-	7.05	**0.84**	2.14	-	10.88	**1.32**	4.41	-	6.01	**0.74**	2.91			
**Min**	2.08	0.1	0.47	-	2.67	0.63	0.45	-	1.79	0.5	0.54	-	0.99	0.34	0.83	-	-	-
**Max**	32.8	2.42	5.20	-	17.6	1.26	5.20	-	20.4	2.51	6.10	-	35.4	1.59	6.10	-	-	-
**St Error**	2.46	0.18	0.42	-	1.24	0.05	0.48	-	1.63	0.16	0.52	-	2.32	0.10	0.60	-	-	-
**Median**	3.755	0.69	0.95	-	5.26	0.82	1.16	-	9.27	1.17	5.00	-	4.41	0.7	1.35	-	-	-
**Mode**	3.86	0.69	0.78	-	-	-	-	-	-	-	5.90	-	-	0.54	6.10	-	-	-
**St deviation**	9.22	0.69	1.57	-	4.64	0.19	1.80	-	6.11	0.58	1.95	-	8.69	0.36	2.24	-	-	-
**Variance**	85.02	0.48	2.45	-	21.5	0.04	3.23	-	37.36	0.34	3.82	-	75.4	0.13	5.00	-	-	-

Background or Reference Soil Sample values: Fe = 4.9, Cu = 0.3, Pb = 0.45

Average enrichment factor (EF) of iron in the soil near SCFPP was 3.35, which classify the soil as “moderately enriched soil” (Table 3). Average I_geo_ value (-0.40) recorded near SCFPP classified the soil as partially uncontaminated. Average CF value of soil around SCFPP was 1.62, which classify the soil as “moderately contaminated soil”. Similarly, average PLI values around SCFPP classify the soil as “moderate degree of pollution”. Average Ecological Risk Index ($E_{r}^{i}$)values for the Iron remained <40 at all distances, which shows low risk for this metal.

**Table 3:** Iron pollution indices around SCFPP.

Distance	EF	I_geo_	CF	PLI	$E_{r}^{i}$
10km	4.14^f^	-0.58^c^	1.58^e^	1.01^b^	1.58^g^
20km	2.22^f^	-0.31^c^	1.44^e^	1.21^b^	1.44^g^
30km	2.57^f^	0.29^d^	2.22^e^	1.84^b^	2.22^g^
40km	4.47^f^	-1.02^c^	1.23^e^	0.74^a^	1.23^g^
**Average**	3.35	-0.40	1.62	1.20	1.62^g^
**Range**	2.22–4.47	-1.02–0.29	1.23–2.22	0.74–1.84	1.23–2.22
**Average Classification**	Moderate Enrichment	Partially Uncontaminated	Moderate Contamination	Moderate Degree of Pollution	Low Risk

^a^: Low degree of pollution,

^b^: Moderate degree of pollution,

^c^: Partially uncontaminated,

^d^: Uncontaminated to moderately contaminated,

^e^: Moderate contamination factor,

^f^: Moderate Enrichment,

^g^: Low risk

About 11 replicates around SCFPP at various distances have shown significantly higher concentration of Fe (upto 35.4 ppm) above the background value of 4.9 ppm (Table 4). EF index classified the soil at these sites as “minimal enrichment” and “moderate enrichment” respectively. I_geo_ values have classified the soil at these sites as “moderately contaminated” and “moderate to heavily contaminated” soils. However, CF index has classified the soil at these sites as “very high contamination”. Ecological Risk Index ($E_{r}^{i}$) values for Iron at each of these highly contaminated sites were recorded <40, which shows low risk.

**Table 4:** Samples with significantly higher concentration of Iron.

Distance	Replicate No.	Concentrations (ppm)	EF	I_geo_	CF	$E_{r}^{i}$
**0–10 km**	A1	18.9	2.38^b^	1.36^c^	3.86^e^	3.86^g^
	A2	19.2	2.42^b^	1.39^c^	3.92^e^	3.92^g^
**11–20 km**	B4	14.8	1.87^a^	1.01^c^	3.02^e^	3.02^g^
	B12	17.6	2.22^b^	1.26^c^	3.59^e^	3.59^g^
**21–30 km**	C4	15.3	1.93^a^	1.06^c^	3.12^e^	3.12^g^
	C6	15.5	1.96^a^	1.08^c^	3.16^e^	3.16^g^
	C9	16.4	2.07^c^	1.16^c^	3.35^e^	3.35^g^
	C11	19.9	2.51^a^	1.44^c^	4.06^e^	4.06^g^
	C13	15.2	1.92^a^	1.05^c^	3.10^e^	3.10^g^
	C14	20.4	2.57^a^	1.47^c^	4.16^e^	4.16^g^
**31–40 km**	D6	35.4	4.47^a^	2.25^d^	7.22^f^	7.22^g^

^a^: Minimal Enrichment,

^b^: Moderate Enrichment

^c^: Moderately contaminated,

^d^: Moderate to heavily contaminated,

^e^: Considerable Contamination,

^f^: Very High Contamination,

^g^: Low risk

### Copper intensity

The mean concentration of Cu in 0–10, 11–20, 21–30, and 31–40 kilometer distance was 0.81 ppm, 0.84 ppm, 1.32 ppm, and o.74 ppm respectively, which is higher than background reference value of 0.3 ppm (Table 2). Average concentration around SCFPP from 0–40 km was 0.93 ppm, much higher than reference value. Values recorded for Iron pollution indices also indicate increased pollution near SCFPP compared to background soil. Average value of PLI (2.75), I_geo_ (0.82), CF (3.09), and EF (4.01) classified the soil as “high degree of pollution”, “Uncontaminated to moderately contaminated”, “Considerable Contamination”, and “Moderate Enrichment” respectively (Table 5). Values of pollution indices recorded in each distance category have shown that the soil of 21–30 km distance was most contaminated. Ecological Risk Index values for Copper was recorded <40, which shows low risk.

**Table 5:** Copper pollution indices near SCFPP.

Distance	PLI	I_geo_	CF	EF	$E_{r}^{i}$
0-10km	1.98^a^	0.40^d^	2.70^f^	4.99^h^	13.5^j^
11-20km	2.72^b^	0.86^d^	2.79^f^	2.60^h^	13.95^j^
21-30km	4.02^c^	1.42^e^	4.40^g^	5.17^i^	22^j^
31-40km	2.26^b^	0.59^d^	2.4^f^	3.28^h^	12^j^
**Average**	2.75	0.82	3.09	4.01	15.45^j^
**Range**	1.98–4.02	0.40–1.42	2.48–4.40	2.60–5.17	12–22^j^
**Average Classification**	High Degree of Pollution	Uncontaminated to moderately contaminated	Considerable Contamination Factor	Moderate Enrichment	Low Risk

^a^: Moderate degree of pollution,

^b^: High Degree of Pollution,

^c^: Very high degree of pollution,

^d^: Uncontaminated to moderately contaminated,

^e^: Moderately contaminated,

^f^: Moderate contamination,

^g^: Considerable Contamination,

^h^: Moderate Enrichment,

^i^: Significant Enrichment,

^j^: Low Risk

At ten replicate sampling sites from 0–40 km distance from SCFPP, Cu concentration was considerably high compared to background value (Table 6). EF index classified the soil at these sites as “moderate enrichment” and “significant enrichment”. I_geo_ values have classified the soil at these sites as “moderately contaminated” and “moderate to heavily contaminated” soils. The CF have classified these selected soils as “very high contamination” class. Ecological Risk Index ($E_{r}^{i}$) values for Copper at sites A2 and C3 have shown moderate risk, while all others site have shown low risk.

**Table 6:** Samples with significantly higher concentration of Copper.

Distance	Replicate No.	Concentrations (ppm)	EF	I_geo_	CF	$E_{r}^{i}$
**0–10 km**	A2	2.42	4.99^a^	2.43^d^	8.07^f^	40.35^g^
	A8	2.26	4.66^a^	2.33^d^	7.53^f^	37.65^h^
**11–20 km**	B4	1.07	2.20^a^	1.25^c^	3.57^e^	17.85^h^
	B8	1.26	2.60^a^	1.49^c^	4.20^e^	21^h^
**21–30 km**	C1	1.62	3.34^a^	1.85^c^	5.40^e^	27^h^
	C3	2.43	5.01^b^	2.43^d^	8.10^f^	40.5^g^
	C10	1.55	3.19^a^	1.78^c^	5.17^e^	25.85^h^
	C12	2.51	5.17^b^	2.48^d^	8.37^f^	41.85^g^
**31–40 km**	D1	1.33	2.74^a^	1.56^c^	4.43^e^	22.15^h^
	D6	1.59	3.28^a^	1.82^c^	5.30^e^	26.5^h^

^a^: Moderate Enrichment

^b^: Significant Enrichment,

^c^: Moderately Contaminated,

^d^: Moderately to Heavily Contaminated,

^e^: Considerable Contamination,

^f^: Very High Contamination Factor,

^g^: Moderate Risk,

^h^: Low Risk

### Lead intensity

The average Lead (Pb) concentration within the 10, 20, 30, and 40 km distance of SCFPP was 1.77 ppm, 2.14 ppm, 4.41 ppm, and 2.91ppm respectively (Table 7). Average concentration of Pb from 0–40 km was 2.81 ppm, much higher than reference value of 0.45 ppm. Values recorded for Pd pollution indices show increased pollution near SCFPP compared to background soil. Average values calculated for the PLI (4.79), I_geo_ (1.56), CF (6.16), EF (7.76) indices from 0–40 km distance have classified the soil as “very high degree of pollution”, “moderately contaminated”, “very high contamination”, and “significant enrichment” respectively. For Lead moderate risk was recorded from 21–30 km distance, while for other distance categories low risk was recorded for this metal.

**Table 7:** Lead pollution indices near SCFPP.

Distance	PLI	I_geo_	CF	EF	$E_{r}^{i}$
0-10km	2.86^a^	0.93^d^	3.92^i^	7.14^k^	19.6^l^
11-20km	3.42^a^	1.19^e^	4.76^i^	7.14^k^	23.8^l^
21-30km	8.13^c^	2.44^f^	9.79^j^	8.38^k^	48.95^m^
31-40km	4.77^b^	1.67^e^	6.16^j^	8.38^k^	30.8^l^
**Average**	4.79	1.56	6.16	7.76	30.8^l^
**Range**	2.86–8.13	0.93–2.44	3.92–9.79	7.14–8.38	19.6–48.95
**Average Classification**	Very high degree of pollution	Moderately Contaminated	Very high contamination factor	Significant Enrichment	Low to Moderate risk

^a^: High degree of pollution,

^b^: Very high degree of pollution,

^c^: Extreme high degree of pollution,

^d^: Uncontaminated to moderately contaminated,

^e^: Moderately Contaminated,

^f^: Moderately to heavily contaminated,

^i^: Considerable contamination factor,

^j^: Very high contamination factor,

^k^: Significant enrichment,

^l^: Low risk

^m^: Moderate risk

There were 26 replicate sampling sites where Pb concentration was significantly higher than the background value of 0.45 ppm (Table 8). Lead concentration recorded at these sites was up to 6.10 ppm at some sites, where calculated EF was up to 8.38. At majority of these sites, EF classified the soil as “significant enrichment”. Similarly, I_geo_ classification classified these soils as “moderately to heavily contaminated” soils, and according to the CF values, the soil at all of these sites have shown “very high contamination factor”. Ecological Risk Index ($E_{r}^{i}$) values for Lead at a number of sites in all distance categories have shown moderate risk.

**Table 8:** Samples with significantly higher concentration of Pb.

Distance	Replicate No.	Concentrations (ppm)	EF	I_geo_	CF	$E_{r}^{i}$
**0–10 km**	A1	3.30	4.53^a^	2.29^c^	7.33^e^	36.65^f^
	A2	4.20	5.77^b^	2.64^c^	9.33^e^	46.65^g^
	A3	5.20	7.14^b^	2.95^c^	11.56^e^	57.8^g^
	A8	3.50	4.81^a^	2.37^c^	7.78^e^	38.9^f^
**11–20 km**	B4	5.20	7.14^b^	2.95^c^	11.56^e^	57.8^g^
	B6	5.00	6.87^b^	2.89^c^	11.11^e^	55.55^g^
	B8	4.00	5.50^b^	2.57^c^	8.89^e^	44.45^g^
	B11	4.70	6.46^b^	2.80^c^	10.44^e^	52.2^g^
	B12	2.80	3.85^a^	2.05^c^	6.22^e^	31.2^f^
**21–30 km**	C3	4.00	5.50^b^	2.57^c^	8.89^e^	44.45^g^
	C4	6.10	8.38^b^	3.18^d^	13.56^e^	67.8^g^
	C5	5.00	6.87^b^	2.89^c^	11.11^e^	55.55^g^
	C6	4.50	6.18^b^	2.74^c^	10.00^e^	50^g^
	C7	4.50	6.18^b^	2.74^c^	10.00^e^	50^g^
	C9	5.90	8.11^b^	3.12^d^	13.11^e^	65.55^g^
	C10	5.70	7.83^b^	3.08^d^	12.67^e^	63.35^g^
	C11	5.00	6.87^b^	2.89^c^	11.11^e^	55.55^g^
	C12	5.90	8.11^b^	3.12^d^	13.11^e^	65.55^g^
	C13	5.90	8.11^b^	3.12^d^	13.11^e^	65.55^g^
	C14	6.10	8.38^b^	3.18^d^	13.56^e^	67.8^g^
**31–40 km**	D3	6.10	8.38^b^	3.18^d^	13.56^e^	67.8^g^
	D4	4.00	5.50^b^	2.57^c^	8.89^e^	44.45^g^
	D5	5.40	7.42^b^	3.00^d^	12.00^e^	60^g^
	D6	6.10	8.38^b^	3.18^d^	13.56^e^	67.8^g^
	D8	6.10	8.38^b^	3.18^d^	13.56^e^	67.8^g^
	D9	4.00	5.50^b^	2.57^c^	8.89^e^	44.45^g^

^a^: Moderate Enrichment,

^b^: Significant Enrichment,

^c^: Moderately to Heavily Contaminated,

^d^: Heavily Contaminated,

^e^: Very High Contamination Factor,

^f^: Low Risk,

^g^: Moderate Risk

Average and individual PERI values for all distance categories and all individual replicate sites were <150 with shows low risk for all metals under study (Table 9).

**Table 9:** PERI values at each replicate sampling site near SCFPP.

Distance	Replicate	PERI	Distance	Replicate	PERI
**0–10 km**	A1	48.7	**21–30 km**	C1	42.1
	A2	90.9		C2	14.7
	A3	74		C3	86.4
	A4	13.7		C4	89.6
	A5	16.3		C5	72.6
	A6	9		C6	76.8
	A7	7.9		C7	69.1
	A8	83.2		C8	25
	A9	23		C9	86.7
	A10	26.2		C10	90.7
	A11	22.3		C11	77
	A12	24.8		C12	109.6
	A13	21.8		C13	92
	A14	23.7		C14	92.3
	Average	34.7		Average	73.17
**11–20 km**	B1	22.9	**31–40 km**	D1	35.5
	B2	18.8		D2	24.3
	B3	23.5		D3	79.8
	B4	78.6		D4	57.2
	B5	30.1		D5	74.4
	B6	74.8		D6	101.5
	B7	24.4		D7	28.9
	B8	67		D8	81.1
	B9	22.2		D9	61.7
	B10	18.7		D10	23.1
	B11	70.9		D11	17.8
	B12	46.9		D12	16.4
	B13	20.6		D13	21.9
	B14	29.2		D14	19.2
	Average	39.19		Average	44.03

The composite soil samples from 0–10, 21–30, and 31–40 km distance were analyzed for heavy metals by using inductively coupled plasma (ICP-OES) (Table 10). Metals including Al, Ba, Mo, Ni, Co and Sr have shown gradual increase in concentration with increase in distance from SCFPP. Concentration of other metals (As, Cr, Se, and Zn) did not show any clear pattern from near to distant soils. Highest Cadmium metal concentration was recorded near the SCFPP compared to distant soils. Statistical analysis shows that overall this trend of variations in the metals concentrations from nearer to distant soils is statistically significant (P-value 0.00) (Table 10).

**Table 10:** Heavy metal contents (ppm) of composite samples near SCFPP.

**Metal**	**Composite sample (0–10 km distance) (ppm)**	**Composite sample (21–30 km distance) (ppm)**	**Composite sample (31–40 km distance) (ppm)**	**Average Concentration (ppm)**
**Al**	0.07	0.12	0.19	0.13
**As**	0.05	0.04	0.06	0.05
**Ba**	0.35	0.42	0.59	0.45
**Cd**	0.09	0.02	0.02	0.04
**Co**	0.12	0.36	0.32	0.27
**Cr**	0.01	0.01	0.01	0.01
**Mn**	18.94	36.11	27.28	27.44
**Mo**	0.02	0.03	0.04	0.03
**Ni**	0.29	0.34	0.35	0.33
**Se**	0.02	0.03	0.02	0.02
**Sr**	2.21	4.34	6.16	4.24
**Zn**	2.08	1.58	3.81	2.49

TWO-WAY ANOVA					
**Source**	**DF**	**Sum of Square (SS)**	**Mean Square (MS)**	**F Statistic (df**_**1**_ **,df**_**2**_ **)**	**P-value**
Factor A	11	2018.0114	183.4556	28.5445 (11,22)	**0.00**
Factor B	2	16.6 829	8.3415	1.2979 (2,22)	0.2932
Error	22	141.394	6.427		
**Total**	**35**	**2176.0883**	**62.174**		

DF-Degree of Freedom

**Fig 3:**
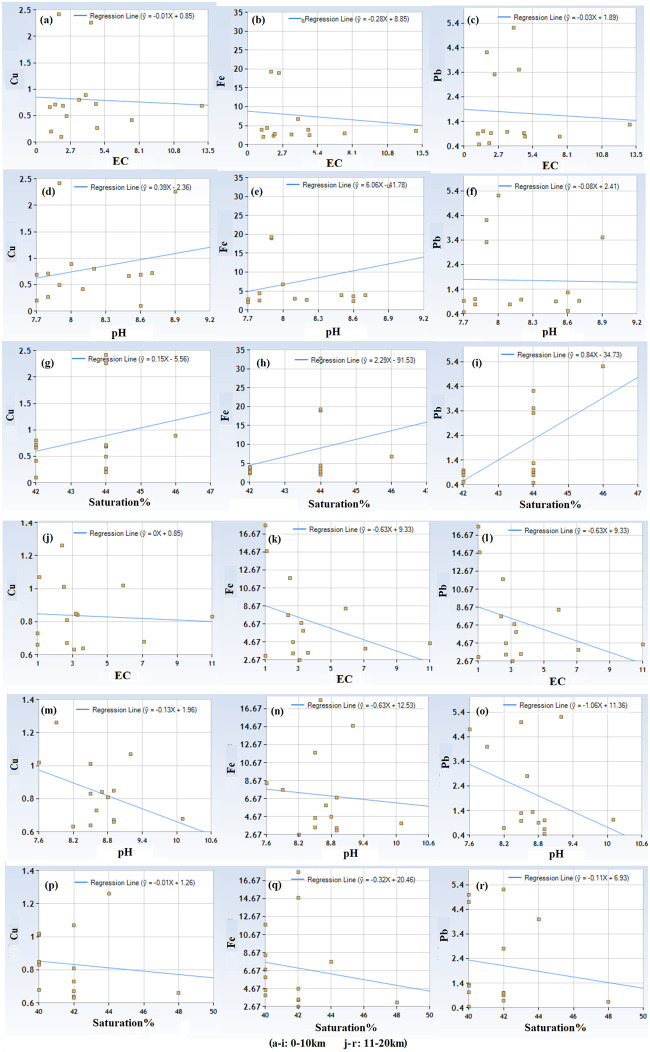
Linear regression equations between physicochemical properties and metals in soils samples in 0–10 km and 11–20 km distance of SCFPP.

**Fig 4:**
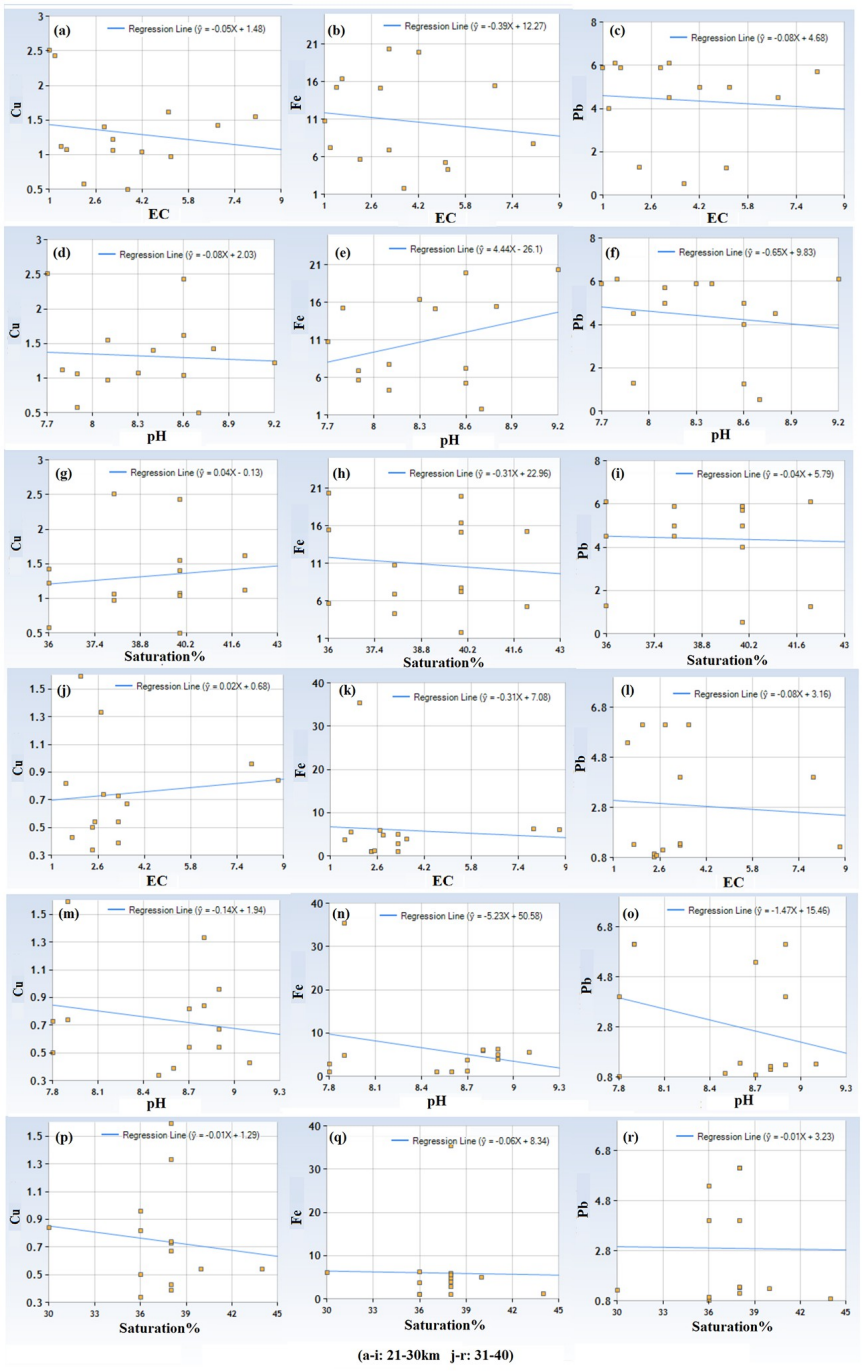
Linear regression equations between physicochemical properties and metals in soils samples in 21–30 km and 31–40 km distance of SCFPP.

Physicochemical parameters including EC, pH, saturation % and texture are given in Table 11. Majority of the soil samples were in the textural class of loam, except few where soil was clay loam and sandy loam. EC, pH, and saturation % of soil samples were ranged between 1–13 dS/m, 7.6–10.1, and 30–48% respectively. Results of the multiple linear regression indicated that there was a weak collective non-significant effect between the Physico-chemical parameters of soil (EC, pH, Saturation %) and metals (Fe, Cu, Pb) in soils around SCFPP at almost all distances except Pb in very vicinity of the SCFPP (Table 12). Multiple linear regression indicated that there was a strong collective significant effect between Physico-chemical parameters of soil (EC, pH, Saturation %) and Pb (Regression equation Pb = -34.727059 + 0.840294 Saturation %, p-value 0.01). A moderate impact of physicochemical parameters of soil (EC, pH, Saturation %) and Cu (Regression equation Cu = 1.962765–0.13008 pH) has also been recorded in 11–20 km distance. However, this moderate link statistically was insignificant (p-value 0.15). Linear regressions among each physicochemical parameter and each metal are in show in Figs 3 and 4.

**Table 11:** Physico-chemical parameters of soil samples.

D	Rep	EC	pH	S%	T	D	Rep	EC	pH	S%	T
**0–10 km**	A1	2.4	7.9	44	L	**21–30 km**	C1	5.1	8.6	42	L
	A2	1.8	7.9	44	L		C2	3.7	8.7	40	L
	A3	3.9	8	46	CL		C3	1.2	8.6	40	L
	A4	4.8	7.8	44	L		C4	1.4	7.8	42	L
	A5	7.5	8.1	42	L		C5	5.2	8.1	38	L
	A6	1.2	7.7	44	L		C6	6.8	8.8	36	L
	A7	2	8.6	42	L		C7	3.2	7.9	38	L
	A8	4.3	8.9	44	L		C8	2.2	7.9	36	L
	A9	4.7	8.7	42	L		C9	1.6	8.3	40	L
	A10	13	8.6	44	L		C10	8.1	8.1	40	L
	A11	2.1	7.7	44	L		C11	4.2	8.6	40	L
	A12	3.4	8.2	42	L		C12	1	7.7	38	L
	A13	1.1	8.5	42	L		C13	2.9	8.4	40	L
	A14	1.5	7.8	44	L		C14	3.2	9.2	36	L
	Mean(±SD)	3.84(±3.17)	8.17(±0.41)	43.43(1.22)			Mean(±SD)	3.56(±2.14)	8.34(±0.44)	(39±2.04)	
**11–20 km**	B1	2.7	8.9	42	L	**31–40 km**	D1	2.7	8.8	38	L
	B2	1	8.9	48	CL		D2	3.3	8.9	40	L
	B3	7.1	10.1	40	L		D3	3.6	8.9	38	L
	B4	1.1	9.2	42	L		D4	3.3	7.8	38	L
	B5	3.3	8.7	40	L		D5	1.5	8.7	36	L
	B6	2.5	8.5	40	L		D6	2	7.9	38	L
	B7	2.7	8.8	42	L		D7	8.8	8.8	30	SL
	B8	2.4	7.9	44	L		D8	2.8	7.9	38	L
	B9	3.6	8.5	42	L		D9	7.9	8.9	36	L
	B10	3.1	8.2	42	L		D10	1.7	9.1	38	L
	B11	5.9	7.6	40	L		D11	2.4	7.8	36	L
	B12	1	8.6	42	L		D12	2.4	8.5	36	L
	B13	3.2	8.9	40	L		D13	3.3	8.6	38	L
	B14	11	8.5	40	L		D14	2.5	8.7	44	L
	Mean(±SD)	3.61(±2.73)	8.66(±0.59)	41.71(±2.20)			Mean(±SD)	3.44(±2.18)	8.52(±0.46)	37.43(±2.98)	

D-Distance, EC-Electrical conductivity (dS/m), S%-Saturation percentage, T-Texture, L-Loam, CL-Clay loam, SL-Sandy Loam, SD-Standards Deviation

**Table 12:** Multiple linear regression equations between Physico-chemical parameters of soil and metals.

**Metal/ distance**	**0-10km (Regression equations and p-values)**	**11-20km (Regression equations and p-values)**
Fe	Fe = -91.526765 + 2.286324 Saturation % (p-value 0.29)	Fe = 9.328932–0.631127 EC (p-value 0.19)
Cu	Cu = -5.558824 + 0.146618 Saturation % (p-value 0.37)	Cu = 1.962765–0.13008 pH (p-value 0.15)
Pb	Pb = -34.727059 + 0.840294 Saturation % (p-value 0.01)	Pb = 11.361831–1.06402 pH (p-value 0.22)
**Metal/ distance**	**21-30km (Regression equations and p-values)**	**31-40km (Regression equations and p-values)**
Fe	Fe = -26.097704 + 4.436314 pH (p-value 0.27)	Fe = 50.579722–5.230395 pH (p-value 0.33)
Cu	Cu = 1.481305–0.045146 EC (p-value 0.57)	Cu = 0.692011 + 0.016179 EC (p-value 0.70)
Pb	Pb = 9.827154–0.650387 pH (p-value 0.62)	Pb = 15.457941–1.473019 pH (p-value 0.29)

## Discussion

In the current study, Copper concentration in the soil samples around SCFPP was found to be higher compared to the background value. At some replicate sites it was calculated to be significantly high. Cu concentration of about 2.51 ppm is quite alarming situation as it is considered to be highly contaminated soil. The mean value of Cu increased up to 20 km of SCFPP, however from 21–30 km, its concentration increased significantly, probably due to the urban settlements and industrial vicinity that include cotton, plastic, agricultural machinery manufacturing, and chemical industry. Various studies reported that industries can increase copper contamination in soil [42, 43]. It was also reported that sources like phosphate fertilizers, metallurgical activities, and mining activities are huge source of copper in soil [43]. From 31–40 km the copper intensity again harmonized with 0–20 km distance. In the current study, in all the distance categories, although the mean values of Copper were significantly higher than the background value, yet they are less than many parts of the world [43–45]. Average concentration of Cu in Nigeria near a coal fired power plant was about 12 ppm [44], 19.6 ppm in Turkey [43], and 39.2 ppm in Serbia [45]. Although these values are higher than the values around CFPP, however these were lower with respected to their country’s background values. Hence in the current case, the elevated Cu concentration can be attributed to CFPP, rather than parent rocks. Past studies conducted in Nikola Tesla CFPP Serbia, the EC, I_geo_, CF and PLI for copper was reported as 1.8, -0.1, 1.4, and 1.2 respectively [35]. The enrichment of copper in the soil around Nikola Tesla CFPP Serbia was deficient to minimal enrichment and the geoaccumultion index showed partially uncontaminated class of soil [35]. Whereas study conducted in India reported the soil to be uncontaminated to moderately contaminated according to geoaccumulation index [46]. The mean contamination factor in Serbia was found to be greater than 1 which showed that the soil was polluted by the copper. Similarly as the pollution load index was 1.2, this showed that the soil samples were poorly contaminated [35]. PLI values have classified the soils around SCFPP as “high degree of pollution”, which need to be further studies. However, the risk reflected from PLI values is not equally complemented by ($E_{r}^{i}$) evaluation. Average Ecological Risk Index ($E_{r}^{i}$) values for Copper classified the soils around SCFPP in all distances a “low risk soil”. However, soils at replicate sites A2 (0–10 km) and C3 (21–30 km) are classified as “moderate risk” soils. The additional input pathways for contamination of Copper at these particular sites need to be investigated and stopped to save these soils from further deterioration. If the impacts of current levels of contamination in these soils are tangible on flora, fauna and humans, remedial measures like bioremediation are suggested.

Lead concentration around SCFPP was significantly higher compared to its background value. At some sites it was calculated to be as high as 6.10 ppm, which is quite alarming situation as it is considered to be highly contaminated soil. This elevated concentration can be attributed to coal fly ash settling within 0–10 km of SCFPP. Apparently, there was no anthropogenic activity or urban settlements other than SCFPP observed within this distance. Similarly comparing to 0–10 km, lead concentration was higher within 11–20 km distance. It was observed that there were no urban settlements or other source present within this distance, except for SCFPP. It slightly increased as the samples collected further 10 km away from the power plant that is about 20 km. Within 21–30 km away from CFPP, lead mean value considerably increased, which may be due to the urban settlements and industrial vicinity like cotton, plastic, agricultural machinery manufacturing, and chemical industry. Whereas in the samples collected from 31–40 km, the lead concentration started to harmonize with concentration in 0–20 km. There may be some other factors that might have impacted the soil. Fuel consumption, farmyard manure, and sewerage slug application has been reported to be sources of lead pollution in soil and agricultural lands [42]. Such anthropogenic activities around SCFPP can contribute to variation in soil heavy metal contents. Studies conducted in Nigeria reported the average concentration of Pb as 138 ppm, which was higher than soil background value of the country [44]. Whereas in Turkey, the lead concentration observed was 19.6 ppm, however this concentration was lower with respect to background values [43]. The EF, I_geo_, CF and PLI for lead in Nikola Tesla CFPP Serbia were found to be 1.3, -0.6, 1.1, 1.2 respectively [35], which were considerably lower compared to current average values of 7.76, 1.56, 6.16, 4.79 for EF, I_geo_, CF and PLI respectively. All these indices classified the soils around SCFPP from “moderately contaminated” to “very high degree of pollution”, which is alarming and require further research to evaluate its impacts on flora and fauna. Average ecological risk index for Lead in 21–30 km distance was recorded as “moderate risk”, while for all other distances it was “low risk”. However, Ecological Risk Index ($E_{r}^{i}$) values for Lead at a number of replicate sites in all distance categories have shown “moderate risk”. Input pathways for this toxic metal especially SCFPP need further assessment to curb this pollution. Remedial options are suggested for highly contaminated sites after thorough assessment.

Elevated concentration of iron can be apparently attributed to coal fly ash settling within the 0–40 km distance of SCFPP, because its concentration is significantly higher than the background values and apparently, there was no anthropogenic activity or urban settlements observed within this area except 21–30 km area. Its concentration slightly decreased as the distance increases from 0–40 km distance. Unusually higher concentration against this pattern within 21–30 km distance may be due to the heavy industries zone within this area with various industries like cotton industry, plastic industry, agricultural machinery manufacturing, and chemical industry. Iron concentration present in soil samples was quite alarming. At some replicate sites Iron concentration recorded was 35.4 ppm, which is heavily contaminated. It can be damaging to the environment as CF values calculated at these sites classify the soils as “significantly to very high contamination factor”. The geoaccumultion index showed partially uncontaminated soil. The mean contamination factor was found to be nearly 1 which showed that the soil was polluted by the iron at some degree. Similarly, as the pollution load index was 1.2, this showed that the soil samples were poorly contaminated [35]. Whereas in past studies conducted like in Nikola Tesla CFPP Serbia, the I_geo_, CF and PLI for iron was found to be -0.9, 0.8, 1.2 respectively. Average Ecological Risk Index ($E_{r}^{i}$) values for the Iron were ranged between 1.32–2.22, that is significantly <40 in all the distances. Hence despite higher values of Fe recorded against background values in the current study, the soil is still classified as “low risk” for this metal. Even at individual 11 sites, where Fe concentration in soil was highest, Ecological Risk Index values classified them as “low risk” soils.

Despite high PLI and $E_{r}^{i}$ values of some individual metals like Pb and Cu, average and individual Potential Ecological Risk Index (PERI) for all distance categories and all individual replicate sites classified the soils around SCFPP as “low risk” soils. It shows that overall health risk of these soils is low, therefore the preventive measures need to be taken in this point of time as prevention is cheaper than remedial measures later. A detailed assessment of all the possible trace metals in these soils is needed.

In composite soil samples, heavy metals including Al, As, Ba, Mo, Ni, Sr and Zn have been detected in higher concentrations within 31–40 km distance. In 21–30 km Co, Mn and Se have been detected in elevated concentration compared to all other composite soil samples. However, higher levels of Cd concentration are detected within 0–10 km of SCFPP. There was no clear pattern of the metal concentration near SCFPP. Apparently in the soils in immediate vicinity (0–10 km) of SCFPP, the overall concentration of these metals was relatively low compared to distant soils. The reason for this trend is unknown and need further investigation to assess all possible input pathways for these metals in addition to SCFPP. Statistical analysis have recorded this unexpected pattern as statistically significant (p-value 0.00), which further demands the more research.

Concentration of heavy metals including As, Cd, Co, Cr, Mn, Ni, Zn, Fe, and Cu in soil around coal fired power plants in many parts of world is considerably high compared to soil near SCFPP in Pakistan (Table 13). However elevated levels of Fe, Cu and Pb near SCFFF compared to background values, suggests the buildup of these metals near SCFPP compared to distant lands. Probable reason of less metal contents compared to other coal fired power plants in the world could be the shorter time span (only 5 years) since it is operational. Average arsenic was found to be 3.5 ppm in India, 13 ppm in Mongolia, 26 ppm in China, and 9 ppm in Turkey [11] compared to 0.05 ppm in the current study. Barium (Ba) concentration was within the range of 0.35 ppm– 0.59 ppm. Its average concertation calculated to be 0.45 ppm. Cadmium (Cd) is quite noticeable as it was highest with the 10 km radius of SCFPP. It was within the range of 0.02 ppm– 0.09 ppm with average concertation of 0.04 ppm. Compared to average concentration (0.04 ppm) of cadmium in this study, its average concentration was 1.3 ppm in Serbia and 0.11 ppm in Turkey [43]. Cobalt (Co) concentration was in range of 0.12 ppm– 0.36 ppm with the average concentration of 0.26 ppm, while it was found to be 13 ppm in Serbia, 18 ppm in China and 64 ppm in Turkey [11]. Chromium (Cr) was having average concentration of 0.01 ppm in soil samples around SCFPP. This concentration is again considerably low compared its concentration (32.2 ppm) in Serbia [35]. Manganese (Mn) concentration was in range of 18.94 ppm– 36.11 ppm. Its average concentration was found to be 27.44 ppm in soils adjacent to SCFPP, compared to significantly higher average values with183 ppm and 721 ppm in South Africa and Turkey respectively [11]. Average concentration of Ni (0.35 ppm) in the soils around SCFPP is considerably low compared to Ni contents of 16 ppm in Mongolia and 610 ppm in Turkey. Whereas Zinc (Zn) concentration in the soil samples were in range of 1.58 ppm– 3.81 ppm with the average concentration of 2.49 ppm. In previous research studies Zn average concentration was found to be 52 ppm in Serbia and 21.22 ppm in Turkey [43].

**Table 13:** Comparison of heavy metals contents in soils near SCFPP with other countries.

Metal	Pakistan Current study		India [4]	Serbia [45]	China [48]	Nigeria [44]	South Africa [47]	Mongolia [32]	Serbia [35]	Turkey [11]
	Range (ppm)	Average Concentration (ppm)								
**Al**	0.07–0.12	0.13	-	-	-	-	-	-	-	-
**As**	0.04–0.06	0.05	3.5	0.9				13		9
**Ba**	0.35–0.59	0.45	-	-	-	-	-	-	-	-
**Cd**	0.02–0.09	0.04	-	1.3	-	2.94	-	-	0.2	
**Co**	0.12–0.36	0.27	19	7.4	18	1.13	-	-	13.4	63.9
**Cr**	0.01–0.01	0.01	103	99.7	99	16.9	63	32	32.2	713.2
**Mn**	18.94–36.11	27.44	1263	780	626	41.8	215		610	720.8
**Mo**	0.02–0.04	0.03	-	-	-	-	-	-	-	-
**Ni**	0.29–0.35	0.33	55	40	30	2.91	32	16	55.9	610.1
**Se**	0.02–0.03	0.02	-	-	-	-	-	-	-	-
**Sr**	2.21–6.16	4.24	-	-	-	-	-	-	-	-
**Zn**	1.58–3.81	2.49	170	88	125	715	87	27	79.6	81.8
**Fe**	6.01–10.88	6.17	-	-	-	5460	1836	-	29030	39065
**Cu**	0.74–1.32	0.72	74	39.2	40	12	56	8	18.2	29
**Pb**	0.45–0.83	0.46	23	52	40	138	52	12	24.1	17

The collective impact of all the Physico-chemical parameters of soil studied on metals is weak and insignificant statistically in almost all the soils. Physico-chemical parameters have shown a strong and statistically significant collective impact on Pb in 0–10 km distance. This differential solitary impact of physic-chemical parameters on Pb may be due to the unique geochemical behavior of Pb, chemical speciation, redox sensitivity, affinity with soil component etc. or anthropogenic activities that increased its mobility in response to EC, pH and Saturation %. The regression analysis between each physico-chemical parameter and individual metal has recorded several differential correlations, with confusing pattern of association. Mostly, Cu was negatively associated with pH, weakly negatively with EC, and both weak positive and weak negative with Saturation %. Fe has shown weak negative correlation with EC, Positive and weak negative with pH, and mix with Saturation %. Similar results were observed for Pb. This mix of correlations indicates complex interplay of factors influencing metal concentrations at different sampling sites. Interaction of physico-chemical parameters (EC, pH, Saturation %) with metal (Fe, Cu, Pb) at different sites may have been influenced by unique soil composition, different land uses and pollution sources other than coal fly ash from SCFPP. Multivariate effect of physico-chemical parameters may also have their role in such results. Further research is needed to find out the answer of these questions considering exploration of wide range of environmental factors.

## Conclusion

Topsoil around the SCFPP was polluted with Pb, Cu, and Fe compared to background soil concentration for these metals. Although various pollution indices classified the soil around SCFPP as uncontaminated to highly contaminated soils, Potential Ecological Risk Index (PERI) has classified these soils as “low risk soils”. The concentration of metals including As, Cr, Co, Cd, and Zn was found within satisfactory limits. There is no clear pattern of metal contamination in topsoil and no clear correlation among parameters of topsoil and metal concentrations indicating complex interplay of other factors at different sampling sites. For complete assessment, further research considering comprehensive environmental parameters, trace metals and input pathways is suggested.
